# Handling Detection Limits of Multiplex Lateral Flow Immunoassay by Choosing the Order of Binding Zones

**DOI:** 10.3390/mi14020333

**Published:** 2023-01-28

**Authors:** Anastasiya V. Bartosh, Dmitriy V. Sotnikov, Anatoly V. Zherdev, Boris B. Dzantiev

**Affiliations:** A.N. Bach Institute of Biochemistry, Research Center of Biotechnology of the Russian Academy of Sciences, 119071 Moscow, Russia

**Keywords:** multiparametric assay, rapid tests, immunochromatography, mycotoxins, non-equilibrium interactions

## Abstract

Changes in the limits of detection (LODs) for a multiplex lateral flow immunoassay (LFIA) caused by different locations of the binding zone on the test strips were studied. Due to the non-equilibrium conditions of the immune reactions in LFIAs, their analytical parameters are susceptible to the binding constants of antigen–antibody reactions and assay duration. Consequently, the integration of several tests into one multiplex assay can cause a significant worsening of the sensitivity. In this study, we propose a simple methodology for the determination of the best arrangement of binding zones, which takes into account the binding constants for immunoreagents. LFIAs of four mycotoxins, namely, aflatoxin B1, deoxynivalenol, T-2 toxin, and ochratoxin A, were integrated into a multiplex test strip. An enzyme-linked immunosorbent assay was applied to determine the equilibrium and kinetic constants of the immunoreactants for each analyte. It was found that the arrangement of binding zones with a descending order of the equilibrium association constants was optimal and provided both lower detection limits and a more uniform coloration. The selected position of the binding zones allowed decreasing the LODs down to 2 and 27 times for ochratoxin A and deoxynivalenol, respectively. The proposed approach can be applied to multiplex LFIAs for different analytes.

## 1. Introduction

Lateral flow immunoassay (LFIA) is implemented using immunochromatographic test strips: multicomposites of several membranes with applied immunoreagents. Contact of a test strip with the tested sample initiates the movement of liquid flow along the membranes, followed by the formation of detectable immune complexes [1]. The simple and rapid implementation of LFIAs allows for the wide application of immunochromatographic test systems in medical diagnostics [2], food safety [3], environmental monitoring [4], and agriculture [5]. The test strips can be used for the multiplex analysis of several analytes simultaneously. In this case, they contain several binding zones [6,7,8,9,10]. However, upon the combination of several assays and, consequently, many specific reagents into one test strip, the positions of the binding zones are changed for different analytes compared to individual tests. This leads to changes in the duration of interactions in solution, while the analyte and the labeled antibodies move to the corresponding binding zone. In addition, the flow rate changes upon moving along the test strip [10]. Usually, the studies devoted to multiplex LFIAs do not consider these factors. However, ignoring them can lead to the loss of binding zone coloration and a deterioration in sensitivity.

As a rule, the length of a test strip used for a multiplex LFIA should be the same as for the monoplex type, because its enlargement increases the consumption of regents and testing duration. The multiplex test systems described in the literature can detect up to five analytes simultaneously [11,12]. This means that the corresponding test strips contain up to six binding zones (including the control). Choice of optimal values for such parameters as pH, ionic strength of reaction media, concentrations of used compounds, etc. can be based on common principles in the design of the experimental strategy, such as full factorial and fractional factorial designs (see examples of their successful application for immunoassays in [13,14]). However, these approaches cannot be transferred for choosing the order of the binding zone location, because the distances from the starting point of flow movement cannot be changed independently for each analyte. The point is that, when increasing the distance for one test zone, we should decrease it for another zone, otherwise the zones will be indistinguishable in the assay result estimation. Therefore, possible combinations of test zone orders should be considered and compared. Note that the more reagents that are combined in a multiplex system or the more different multiplex tests that are implemented, the more time-consuming the direct experimental characterization of all options for the location of binding zones. The number of variants for the sequential arrangement of N binding zones is factorial of N (N!). That is, it grows rapidly with increasing N. For example, 5! = 120. It should also be taken into account that the data for tests with a lower quantity of binding zones cannot be used in the design of more complicated tests, due to the different distances of the binding zones. Therefore, simple approaches for evaluating the optimal location of individual reagents on the multiplex test strips are in great demand.

In our previous study, analytical signals (coloration intensities) were compared for different analytes at different positions of the zones on the test strip [10]. However, the reasons behind signal changes were not discussed. In this work, we propose a simple methodology for determining the best arrangement of binding zones, based on the immunochemical characteristics of the reagents. For this, a multiplex LFIA for four mycotoxins, namely, aflatoxin B1 (AFB1), deoxynivalenol (DON), T-2 toxin (T-2), and ochratoxin A (OTA), was considered. These compounds are widespread toxic contaminants of agricultural products [15,16,17,18]. As these analytes are small molecular weight antigens, an LFIA was implemented in the indirect competitive format. Here, conjugates of specific antibodies with gold nanoparticles (AuNPs) as a common LFIA label are changed to a combination of non-modified specific antibodies and anti-species antibodies labeled with AuNPs. Previously, it was demonstrated that the indirect LFIA format prevents non-productive binding of the analyte to specific antibodies, without changes in the detected signal [19,20,21,22,23,24]. In this case, the concentrations of AuNPs and specific antibodies can be varied independently. This enables achieving both high signals caused by a high content of the label and efficient immune competition due to decreased concentration of specific antibodies. Moreover, indirect labeling allows using the same labeled conjugate to generate signals in all binding zones and, thus, eliminates potential differences in the immobilization of different specific antibodies on AuNPs [25].

## 2. Materials and Methods

### 2.1. Reagents and Materials

DON, OTA, AFB1, and T-2 were from Chromresurs (Moscow, Russia). Mouse anti-DON, anti-OTA, anti-AFB1, and anti-T-2 monoclonal antibodies, as well as conjugates of DON, OTA, AFB1, and T-2 with bovine serum albumin (BSA), were purchased from Eximio Biotec (Wuxi, China). Mouse polyclonal immunoglobulins, goat anti-mouse polyclonal immunoglobulins (GAMI), and goat anti-mouse polyclonal immunoglobulins labeled with horseradish peroxidase were purchased from Arista Biologicals (Allentown, PA, USA). Chloroauric acid, sodium citrate, sodium azide, 3,3′,5,5′-tetramethylbenzidine dihydrochloride (TMB), and Tween-20 were from Sigma-Aldrich (St. Louis, MO, USA). BSA was from Boval Biosolutions (Cleburne, TX, USA). Sucrose was from Cristalco (Paris, France). Tris, NaCl, K_2_PO_4_, and KOH were from Chimmed (Moscow, Russia). The ground corn was from Trilogy (Washington, MO, USA).

All solutions were prepared using deionized water (18 MΩ·cm at 25 °C) obtained on a Simplicity Milli-Q system (Millipore, Billerica, MA, USA). Working Hi-Flow Plus 135 nitrocellulose membrane and a glass-fiber membrane PT-R7 of 5 mm width were from MDI (Ambala Cantt, India). A CF5 absorbance membrane was from Whatman (GE Healthcare Bio-Sciences, Marlborough, MA, USA).

### 2.2. Determination of Constants of Immune Interaction Using an Enzyme-Linked Immunosorbent Assay

As a coating antigen in the enzyme-linked immunosorbent assay (ELISA), a hapten–protein conjugate (1 μg/mL, 100 μL) in 50 mM potassium phosphate buffer, pH 7.4 with 0.1 M NaCl (PBS), was added to the microplate wells and incubated for 2 h at 37 °C. After washing three times in PBS with 0.05% Triton X-100 (PBST), 1% BSA solution (100 μL) in PBST was added and incubated for 1 h at 37 °C. After washing, specific antibodies (1 ng/mL—2 μg/mL, 50 μL) in PBST were added. Then, goat anti-mouse antibodies labeled with horseradish (2 μg/mL, 50 μL) were added and incubated for 1 h at 37 °C. After that, the microplate was washed. To compare the signal, an excess amount of the antigen (0.5 μg/mL) was added to half of the microplate wells and incubated for 1 h at 37 °C. To measure the peroxidase activity, a substrate solution (0.42 mM TMB and 1.8 mM H_2_O_2_ in 0.1 M citrate buffer (pH 4.0), 100 µL) was added to the microplate wells. After 15 min incubation at room temperature, the reaction was stopped by adding 1 M H_2_SO_4_ (50 μL). The optical density (OD) of the reaction product was measured at 450 nm using a Zenyth 3100 microplate photometer.

### 2.3. Synthesis of AuNPs and Their Conjugation with GAMI

AuNPs with a diameter of 30 nm were synthesized as described in our previous studies [26]. For this, 0.1 mL of 10% HAuCl_4_ was added to 97.5 mL of deionized water and brought to a boil. After that, 1.5 mL of 1% sodium citrate was added and the resulting mixture was boiled for 25 min. The obtained conjugate was cooled and stored at 4 °C.

To obtain conjugates, GAMI were dialyzed against 10 mM Tris-HCl buffer, pH 8.6. AuNPs (8 mL) with pH adjusted to 8.5 using 0.2 M K_2_CO_3_ were added to GAMI (1 mg/mL, 150 µL) and incubated with stirring for 45 min at room temperature. Then, 200 µL of 10% BSA was added and incubated under stirring for 15 min at room temperature. The obtained conjugate was separated from the unbound GAMI by centrifugation at 10,000× *g* at 4 °C for 15 min. The precipitate was dissolved in 1 mL of PBS containing 0.25% BSA, 0.25% Tween 20, 1% sucrose, and 0.05% NaN_3_ [27]. An OD_520_ of the obtained conjugate was measured using a Libra S60 spectrophotometer (Biochrom, Cambridge, UK).

### 2.4. Preparation of the Test Strips

The DON-BSA, OTA-BSA, AFB1-BSA, and T-2-BSA conjugates (1 mg/mL) and mouse IgG (0.05 mg/mL) were applied to the working membrane (0.1 μL per 1 mm) to form the test and control zones, respectively. The labeled GAMI and specific antibodies (5 µg/mL, 1.2 µg/mL, 7 µg/mL, 0.5 µg/mL for anti-DON, anti-OTA, anti-AFB1, and anti-T-2 monoclonal antibodies, respectively) in PBS containing 0.25% BSA, 0.25% Tween 20, and 1% sucrose were applied to the glass-fiber membrane (32 µL/cm). After applying the reagents, the membranes were dried for 10 h at 37 °C. Then, the multimembrane composite was assembled and cut into test strips of 3.3 mm width using an automatic Index Cutter-1 guillotine (A-Point Technologies, Gibbstown, NJ, USA). The obtained test strips were sealed in foil bags containing silica gel as a desiccant. Cutting and packaging were carried out at 20–22 °C in a special room with a relative humidity of not more than 30%.

### 2.5. LFIA of Mycotoxins

Test strips were taken from the bags, brought to room temperature, and immersed into the samples with various concentrations of mycotoxins (8 samples; 450 ng/mL, 150 ng/mL, 300 ng/mL, 150 ng/mL for DON, OTA, AFB1, and T-2, respectively; dilution 3 times per step) in 50 mM phosphate buffer, pH 7.4, with 0.25% Tween-20 for 7 min [27]. The samples of the ground corn were mixed with an extraction solution (methanol:water, 70:30) in a 1:5 (*v*/*v*) proportion and incubated with gentle stirring at room temperature for 1 h [28]. After centrifugation for 10 min at 8000× *g* at 4 °C, the supernatant was collected and stored at 4 °C. The obtained extracts were spiked with mycotoxins and diluted with PBST (1:2.5, *v*/*v*) before the LFIA.

### 2.6. Data Processing

After the LFIA, images of the test strips were obtained using a scanner (Canon Lide 90, Canon, Tokyo, Japan) under a resolution of 600 dpi without contrasting or color correction. The intensities of coloration were quantified using Total Lab software (TotalLab, Newcastle upon Tyne, UK). The dependence of the color intensity of the test zones (for the LFIA) or OD (for the ELISA) (y) on the analyte concentration (x) was approximated by a four-parameter function using the Origin 7.5 software (Origin Lab, Northampton, MA, USA). The concentrations of analytes that caused inhibition of the binding of the enzyme label to the solid phase (for the ELISA) and the binding of the labeled anti-species antibodies in the test zone (for the LFIA) by 50% and 10% (IC_50_ and IC_10_, respectively) were determined [29]. Visual LOD was estimated as the analyte concentration causing the disappearance of visible coloration in the test zone. The instrumental LOD was considered as the minimum reliably detectable analyte concentration (IC_10_).

## 3. Results and Discussion

### 3.1. Dependence of Signal Intensity on the Location of the Binding Zone in the LFIA

**Figure 1:**
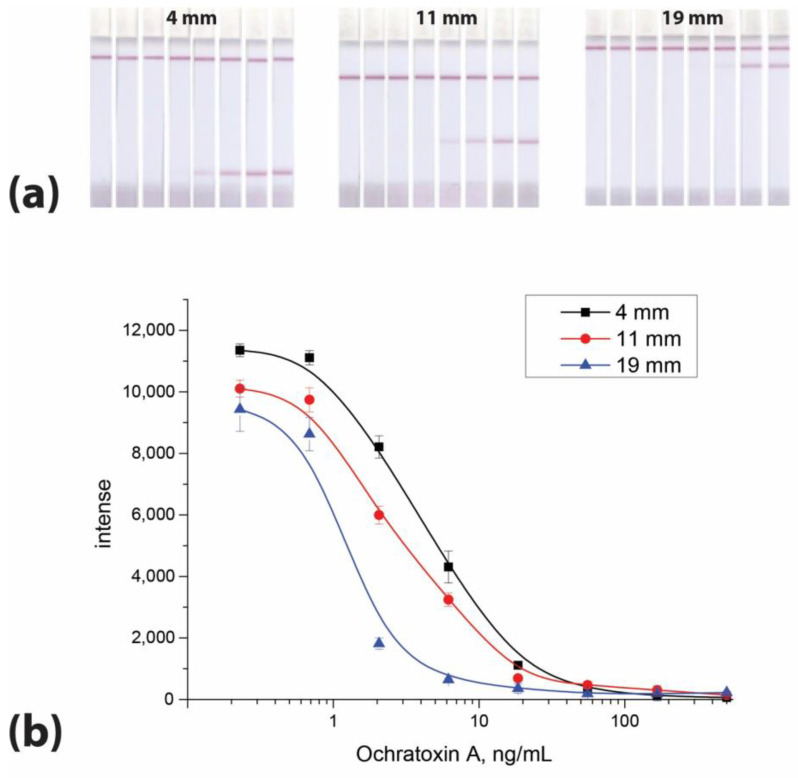
Images of test strips (**a**) and calibration curves of OTA (**b**) in the LFIA with different locations of binding zones (4 mm, 11 mm, and 19 mm from the lower edge of the working membrane, respectively). The test strips are arranged from right to left according to the OTA concentrations of 500, 166, 55.5, 18.5, 6.2, 2.1, 0.7, and 0 ng/mL. The experiments were implemented in triplicate, and standard deviations are given as error bars.

The location of the test zone on the working membrane can affect the analytical parameters of an LFIA [30]. In our previous study, we demonstrated that upon changing the position of the binding zone its coloration and the assay sensitivity also changed [10]. In this study, the same effect was observed for OTA: the OTA LOD in the LFIA depends on the position of the binding zone (Figure 1).

This change can be explained by slow immune interactions and the limited time for the immunoreagents to move to the binding zone located close to the lower edge of the working membrane. This factor seems very important because, in rapid test systems, immune interactions do not often reach equilibrium, while its degree is critical for the number of complexes formed and detected. Changing the flow rate significantly affects assay sensitivity. Therefore, the location of the test zone at the upper edge of the working membrane near the control zone is usually considered the preferred mode [31]. However, this requirement cannot be achieved for all analytes detected in a multiplex analysis. In addition, the concentrations of the interacting reagents change as a result of their dilution upon movement of the flow along the test strip [30,31]. This affects not only the chemical equilibrium in the solution but also the binding efficiency of the label when it reaches the test zone [10]. The location of the test zone as far as possible from the flow start can eliminate these problems. On the other hand, other reagents may have effective immune binding both in solution and with immobilized reagents, regardless of the location of the test zone. In this study, we relied on the immune interaction constants to estimate the difference in the time necessary for reaching equilibrium for all reagents.

### 3.2. Determination of Equilibrium and Kinetic Constants

First, equilibrium association and dissociation constants (K_a_ and K_d_) were determined with the help of ELISA. In contrast to the traditional indirect ELISA, which involves the sequential addition of reagents, incubation, and washing off unbound reagents, we carried out simultaneous incubation of specific antibodies and anti-species labeled antibodies, followed by washing off the unbound reagents. This was performed so that the complex of antibodies and the immobilized antigen could not dissociate during a 1 h incubation with the labeled anti-species antibodies. For this reason, the latter were used at an excessive concentration (2 μg/mL) to detect the formed immune complexes, ensuring maximum label binding to specific antibodies.

**Figure 2:**
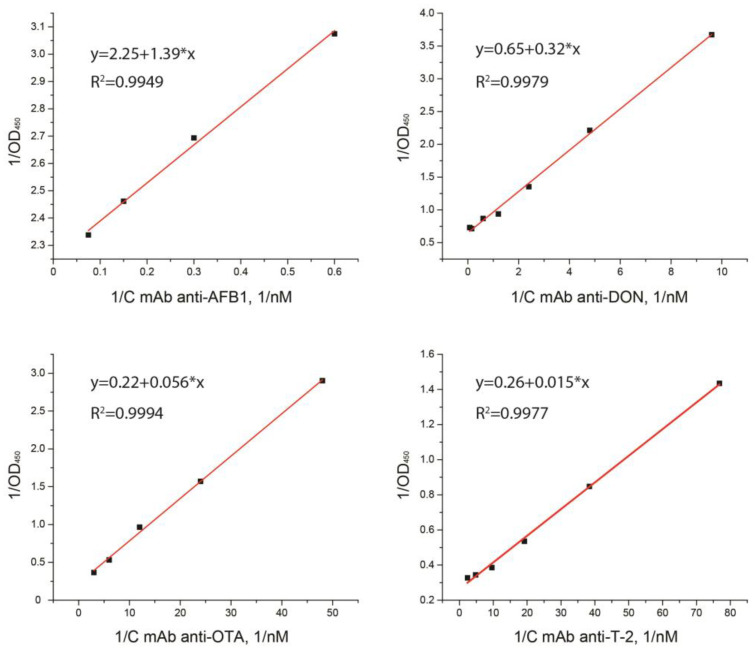
Dependencies of OD450 on antibodies’ concentration in reverse coordinates in the ELISA. 1/OD450 is the inverse signal intensity (*n* = 3) and 1/C is the inverse concentration of antibodies (1/nM).

To determine K_a_ and K_d_, the dependencies of OD_450_ on antibodies’ concentration were obtained and plotted in double inverse coordinates (Figure 2).

Under an excess of antibodies, such dependencies were almost linear. Accordingly, the criterion for the applicability of the described model was the linearizability of the obtained dependencies. To estimate the constants, antibody concentrations for which the R-factor of linearization exceeded 0.9 were selected. This guaranteed that the concentration of antibodies was much higher than the concentration of the immobilized antigen. The straight lines obtained for four analytes allowed calculating the K_d_ and K_a_ values according to the described procedure (see Supplementary Materials). Note that the choice of the concentration of secondary antibodies did not affect the measurement of the constants. A decrease in the binding of mAb to the antigen with an increase in the concentration of secondary antibodies was not observed. The obtained constant values are presented in Table 1. 

**Figure 3:**
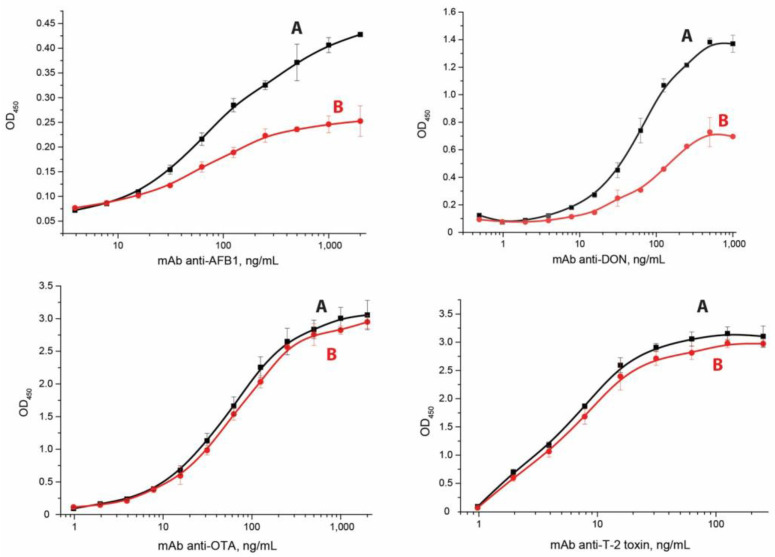
Dependencies of OD_450_ on antibodies’ concentration in reverse coordinates in the ELISA without the addition of the analyte (**A**) and with excess of the analyte (**B**). The experiments were implemented in triplicate, and standard deviations are given as error bars.

The second step was to calculate the association and dissociation kinetic constants of (k_a_ and k_d_). For this, the dependencies of OD_450_ on the concentrations of antibodies upon the addition of the analyte at excess concentrations were obtained (Figure 3). The measurement of kinetic dissociation constants was based on the following principle. Immune complexes of immobilized hapten-BSA conjugate and added antibodies were formed in microplate wells and then, after removal of unbound antibodies, were dissociated. To prevent the re-association of the released antibodies with the hapten-BSA conjugate, an excess of free hapten was added to the wells. Reactant concentrations were chosen to provide this excess. Namely, the hapten-BSA conjugates were added to the wells for immobilization at a concentration of 1 μg/mL (~15 nM per BSA). The amount of protein per well was comparable to the nominal capacity limit of the used microplates, but the number of actually immobilized conjugates could have been less. Although the conjugate contains several hapten groups, steric hindrances limit the amount of antibodies that can bind to one molecule of the immobilized conjugate to a few antibodies. Taking into account these factors, we can state that the content of conjugated hapten in our system is near or higher than the level of 10^-8^ M. AFB1, DON, OTA, and T2 were added at a concentration of 0.5 µg/mL, which corresponded to molar concentrations in the range of 1.1–1.7 µM. Thus, the concentration of free hapten was approximately two orders of magnitude more than the concentration of conjugated hapten. Under these conditions, the binding of dissociated antibodies with free hapten was much more likely than the re-association with conjugated hapten. That is, the dissociation of the antibody-(hapten-BSA) complex in our experiment could be considered an irreversible process, and its rate was determined only by the value of the kinetic dissociation constant (see Supplementary Materials, Section S2).

The curves of anti-OTA and anti-T2 are quite close to each other, due to low kinetic dissociation constants (less than 10^−4^ s^−1^), which suggests a weak dissociation of the formed complexes within the time of the dissociation stage in the conducted experiment (one hour). To check the influence of the incubation time on the dissociation of immune complexes, we performed these processes under a high excess of the competitor (free antigen). As can be seen from the Supplementary Information, Figure S2, the time used in our study accorded to the interval with a rapid decrease of immune complex quantity for all four studied mycotoxins.

The kinetic constants were calculated based on the changes of the analytical signal over time in comparison with the curves without the addition of the analyte (see Supplementary Materials); k_a_ and k_d_ values are presented in Table 1.

### 3.3. Factors Affecting Complexation in the Multiplex LFIA

**Figure 4:**
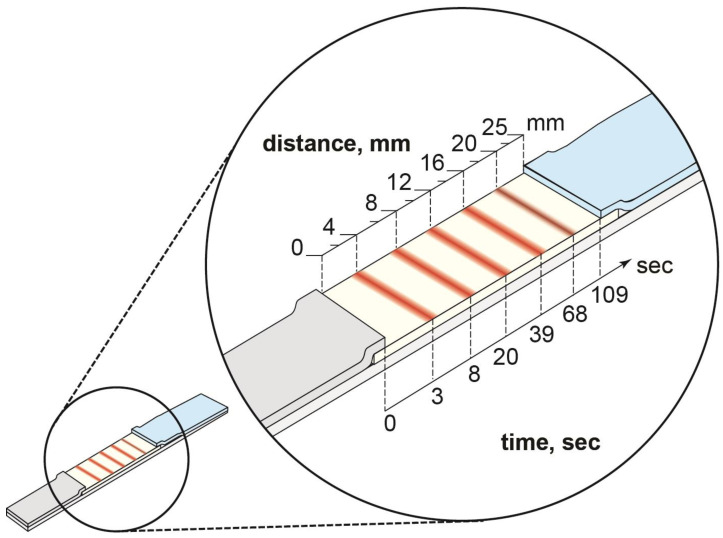
Geometry of the test strip. The distances to the binding zones and the time needed for the flow to reach them are indicated on the left and right sides of the test strip.

The decrease in the flow rate under increasing distance from the flow start was previously demonstrated for a LFIA [32,33]. Due to this, the location of the binding zones affects their coloration intensity and, therefore, the assay sensitivity. The maximum intensity is registered in the absence of the analyte in the sample. In this case, this value depends on the time required for the labeled antibodies to flow along the binding zone (Figure 4).

LFIA may be carried under either a lack of labeled antibodies or their excess. In the first case, labeled antibodies fully move along the binding zone. Therefore, the reaction time in the binding zone will increase with increasing distance from the start to the binding zone because of flow slowing. Under an excess of labeled antibodies, they will flow along the working membrane during the entire time of the assay. In this case, the reaction time in the binding zone decreases with the increasing distance to the binding zone by the duration required for the liquid front to reach it. For multiplex analysis, the second case is relevant because the reactions with all analytes in each binding zone require an excess of labeled antibodies. Therefore, the greater the distance of the test line from the start, the smaller the amount of the complex formed in this zone and the lesser the coloration intensity. In the case of antibodies with a higher affinity, immune complexes form faster, which results in faster signal generation. On the contrary, for low-affinity interactions, an increase in the signal generation time is more critical, due to slower kinetics. Thus, the revealed influence of the binding zone location on the signal amplitude allows for the division of immunoreagents into three groups—those with a minimum preferred distance of the binding zone from the start, a preferred maximum distance, and a reduced influence of distance on the analytical parameters. For this ranking, the interaction constants for all studied pairs of immunoreagents had to be determined. It should be noted that the interaction constants were calculated for the conjugated haptens. These values seem preferable to the binding constant of free haptens because, in the binding zone, the complex is formed due to the interaction of the labeled antibodies with the conjugated hapten.

### 3.4. Multiplex LFIA

**Figure 5:**
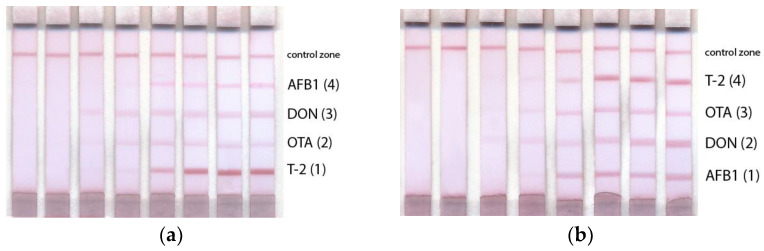
Multiplex test system with test zones arranged in order of ka in reverse (**a**) and the increasing order (**b**). Concentrations of mycotoxins for the 8 samples were 450 ng/mL, 150 ng/mL, 300 ng/mL, 150 ng/mL for DON, OTA, AFB1, and T-2, respectively; dilution 3 times per step. The test strips are arranged from left to right according to the AFB1 concentrations of 300, 100, 33.33, 11.11, 3.70, 1.24, 0.41, 0 ng/mL; DON concentrations of 450, 150, 50, 16.67, 5.56, 1.85, 0.62, 0 ng/mL; OTA concentrations of 150, 50, 16.67, 5.56, 1.85, 0.62, 0.21, 0 ng/mL; T-2 toxin concentrations of 150, 50, 16.67, 5.56, 1.85, 0.62, 0.21, 0 ng/mL.

Based on the regularities and differences found in the properties of the immunoreagents, a multiplex test system was composed, where the location of the binding zones corresponded to ka. Consequently, the binding zones were formed in order of increasing K_a_ and the reverse order (from bottom to top of the test strip): (AFB1 (1) → DON (2) → OTA (3) → T-2 (4) and T-2 (1) → OTA (2) → DON (3) → AFB1 (4) (Figure 5).

**Figure 6:**
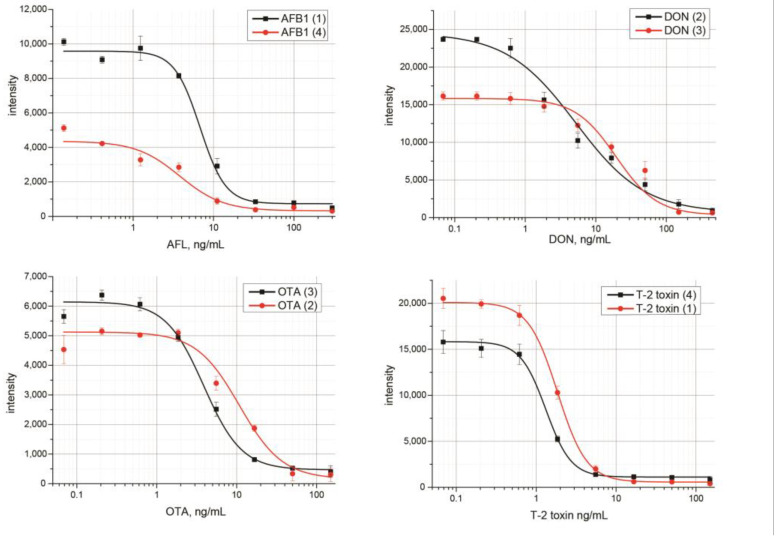
Calibration curves for mycotoxins in the multiplex LFIA. The experiments were implemented in triplicate, and standard deviations are given as error bars.

Figure 5a demonstrates that, in the case of arranging the binding zones in order of decreasing k_a_, the signal values differed greatly. For the immune pair characterized by the least affinity (AFB1), the color intensity was extremely low, which made AFB1 detection difficult. In the case of the test zone arrangement in the order of increasing K_a_, the difference in color intensity was less pronounced (Figure 5b). Therefore, upon the given arrangement of the reagents, the coloration of the binding zones was more uniform, which is preferable for a multiplex LFIA. Figure 6 demonstrates the difference between the signals of the same analytes in different positions on the test strip.

Two reactions take place in competitive LFIA, the reaction of antibodies with a competitor and the reaction of antibodies with hapten–protein conjugate in the test zone. Since the conjugate is immobilized in high concentrations (1 mg/mL, i.e., ~15 nM per BSA), the antibody binding in the test zone proceeds quickly. At the used concentrations of reactants, even for the least affinity pair, the reaction approached 92% of the final product value in 1 sec (see COPASI modeling in Supplementary Information, Figure S3). The time for reaching equilibrium in the antibody–competitor reaction was determined by the competitor’s concentration. At high concentrations, equilibrium is reached during the liquid movement to the test zone. At low concentrations, the kinetic regime continued. For these reasons, both the kinetic and equilibrium parameters of the reactions can influence the final assay result.

As can be seen from the Table 1, for reagents specific to APB1, DON, and T-2, the higher the kinetic association constant, the higher the equilibrium association constant. Only OTA-specific reagents deviate from this dependence: Ka is high due to a low kinetic dissociation constant. Thus, the equilibrium constant is more informative for making decisions about the design of a multiplex test system compared with the kinetic association constant.

Different positions of the binding zones affected the analytical characteristics of the test system. Thus, when the binding zones were located in the order of increasing K_a_ from the bottom to the top of the test strip (AFB1 (1) → DON (2) → OTA (3) → T-2 (4)), an increase in the signal for AFB1, OTA, and DON detection was observed, while the signal for T-2 detection was slightly reduced. With the given arrangement of the reagents, the multiplex test system was characterized by a more uniform coloration of the test zones and sensitive determination when compared with a test system where the reagents were arranged in decreasing order of Ka. In general, the higher the affinity of antibodies, the less the influence of the position of the binding zones on the analytical characteristics and vice versa. 

As noted, the farther the liquid front is from the start, the slower it moves (see the times to reach the test zones in Figure 4) and, accordingly, the time of interaction with the immobilized hapten–protein conjugate increases. Notably, these differences affect the maximum signal level (the degree of antibody binding in the absence of the analyte), while the attenuation of the signal by the reaction with free antigen is determined by the processes in the solution, and not by the speed of crossing the binding zones.

The influence of the position of the zone on the level of antibody binding to it depends on several factors. On the one hand, as the liquid front moves away from the start, its velocity slows down and, accordingly, the time of interaction with the immobilized hapten–protein conjugate increases. On the other hand, as the conjugate flows through the working membrane, it diffuses in the liquid, distributing more evenly in the volume and reducing its concentration. Thus, the concentration of the marker in the lower (starting) part of the strip is higher than in its upper part.

The analytical characteristics of the test systems are presented in Table 2. These parameters are comparable to those for a monoplex systems (see Supplementary Information), were retained when tests were carried out in a grain extract, and the LOD meets practical requirements, as per the recommendations of the European Commission Regulation 1881/2006 [34].

**Figure 7:**
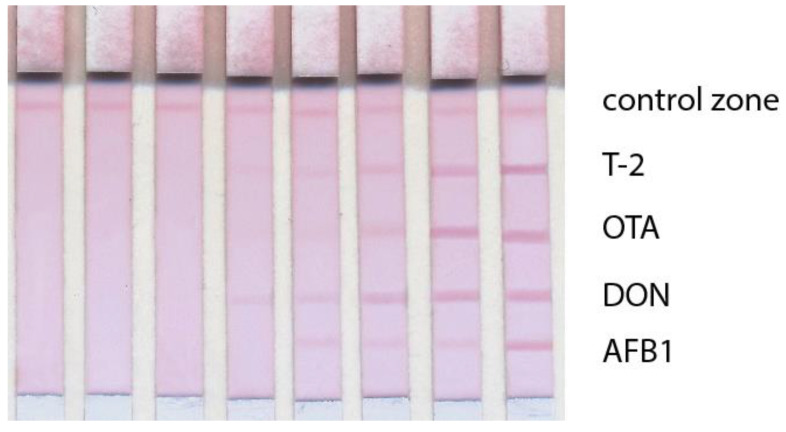
Images of test strips after the LFIA of mycotoxins in corn extracts. Signal intensity dependence on antigen concentration. The test strips are arranged from left to right according to the AFB1 concentrations of 300, 100, 33.33, 11.11, 3.70, 1.24, 0.41, 0 ng/mL; DON concentrations of 450, 150, 50, 16.67, 5.56, 1.85, 0.62, 0 ng/mL; OTA concentrations of 150, 50, 16.67, 5.56, 1.85, 0.62, 0.21, 0 ng/mL; T-2 toxin concentrations of 150, 50, 16.67, 5.56, 1.85, 0.62, 0.21, 0 ng/mL.

The applicability of the developed multiplex LFIA for the detection of mycotoxins in grain samples was tested. For this, corn extracts spiked with AFB1, DON, OTA, and T-2 were analyzed (Figure 7). It was demonstrated that the LODs (converted to g/g) remained the same as upon detection in the buffer and met practical requirements for mycotoxin determination.

## 4. Conclusions

Despite the well-studied theoretical basis of LFIAs, their optimization comes down to the enumeration of a large number of experimental options. This study shows that taking into account the binding constants for immunoreagents is very important when developing multiparametric test systems. This issue is usually ignored and the selection of the best location for the binding zones is missed. The proposed protocol for the optimization of multiparametric test systems developed in this study includes determining the constants for all pairs of immunoreagents. On the basis of this data, the arrangement of the test zones in the order of increasing equilibrium association constant was determined. The proposed method for choosing the location of binding zones in a multiplex LFIA excludes the necessity of the consideration of numerous options. In this way, the LFIA achieves closer color intensities for the test zones and an increased sensitivity of detection for certain analytes. As a result, the developed LFIA is suitable for simultaneous determination of multiple analytes and had the best performance using the given reagents.

## Figures and Tables

**Table 1:** Values of the equilibrium (K_a_, K_d_) and kinetic (k_a_, k_d_) constants.

Analyte	K_a_, M^−1^	K_d_, M	k_a_, M^−1^·s^−1^	k_d_, s^−1^
OTA	4.0 × 10^9^	2.5 × 10^−10^	9.6 × 10^4^	2.4 × 10^−5^
T-2	1.7 × 10^10^	5.8 × 10^−11^	4.0 × 10^5^	2.3 × 10^−5^
AFB1	1.6 × 10^9^	6.2 × 10^−10^	1.7 × 10^5^	1.1 × 10^−4^
DON	2.1 × 10^9^	4.8 × 10^−10^	3.3 × 10^5^	1.6 × 10^−4^

**Table 2:** Analytical parameters of the developed multiplex LFIA for four mycotoxins. The experiments were implemented in triplicate, and standard deviations are given as error bars.

Analyte	Test Strip Design	Position	Instrumental LOD, ng/mL	IC_50_, ng/mL
AFB1	Optimized	1	2.9 ± 0.4	7.6 ± 0.8
AFB1	Non-optimized	4	2.1 ± 0.6	4.3 ± 0.3
T-2	Non-optimized	1	0.7 ± 0.3	1.4 ± 0.3
T-2	Optimized	4	0.8 ± 0.1	1.5 ± 0.1
DON	Optimized	2	0.3 ± 0.1	2.9 ± 1.5
DON	Non-optimized	3	8.1 ± 2.5	19.9 ± 4.0
OTA	Non-optimized	2	2.5 ± 0.3	9.5 ± 1.4
OTA	Optimized	3	1.5 ± 0.6	4.7 ± 1.2

## Data Availability

Data are contained within the article. Initial data of instrumental measurements are available on request from the corresponding author.

## Supplementary Materials

The following supporting information can be downloaded at: https://www.mdpi.com/article/10.3390/mi14020333/s1, S1. Determination of equilibrium constants; S2. Determination of the kinetic dissociation constant; Figure S1: Calibration curves for mycotoxins in the multiplex LFIA; Figure S2: Dynamics of dissociation of the immune complexes.
