# Supplementary material for: Handling Detection Limits of Multiplex Lateral Flow Immunoassay by Choosing the Order of Binding Zones
Source: Micromachines (Basel). 2023 Jan 28;14(2):333. doi: 10.3390/mi14020333 (PMC9965744; doi:10.3390/mi14020333)
Supplement: Supplementary file 1 [file micromachines-14-00333-s001.zip › micromachines-2073058-supplementary.pdf]

Micromachines

Handling detection limits of multiplex lateral flow immunoassay by choosing the order of binding zones

Anastasiya V. Bartosh[1], Dmitriy V. Sotnikov, Anatoly V. Zherdev and Boris B. Dzantiev*

Correspondence: dzantiev@inbi.ras.ru

Supplementary

S1. Determination of equilibrium constants

Complexation of an antigen with an antibody is a reversible bimolecular reaction:

Ab + Ag = AbAg (1).

The equilibrium association constant (Ka) of this reaction is:

$Ka = \frac{[AbAg]}{[Ab][Ag]}$, (2),

where $Ka[Ab][Ag] = [AbAg]$ (3).

Because the concentration of the immobilized antigen during the ELISA is unknown, it is convenient to change the concentration value to the proportion of bound antigens (θ), which is equal to [AbAg]/[Ag]0 (subscript 0 indicates the initial concentration). Under the excess of antibodies, the ratio [Ag]/[Ag]0 is be equal to 1–θ (i.e., the proportion of free antigens). As a result of dividing both parts of equation (3) by [Ag]0 and using θ value, the following equation is obtained:

$Ka[Ab](1 - \theta) = \theta$ (4).

The θ value can be expressed as S/Smax, where S is the detected analytical signal, and Smax is the maximum theoretically possible signal that would be observed if the entire antigen binds to the antibodies. This approach enables calculating constants of the complex formation at an unknown concentration of the immobilized antigen. After replacing θ with S/Smax, equation (4) is transformed into:

$Ka[Ab]\left(1 - \frac{S}{Smax}\right) = \frac{S}{Smax}$ (5).

After transformations and replacement of Ka to Kd (equilibrium dissociation constant of the complex, Kd = 1/Ka), the equation (5) is transformed into:

$\frac{Kd}{[Ab]Smax} + \frac{1}{Smax} = \frac{1}{S}$ (6).

Equation (6) shows that the inverse signal is linearly dependent on the inverse antibody concentration. In this case, if the concentration of added antibodies is much higher than the concentration of the immobilized antigen, [Ab]≈[Ab]0. Therefore, to calculate the equilibrium interaction constant, a dependency of OD_{450} on the antibody concentration is obtained. In the range of antibody concentrations that are much higher than the concentration of the immobilized antigen, a linear dependence of 1/S on

1/[Ab]0 should be observed. For excess antibody concentrations, the linearized dependence of 1/S on 1/[Ab]0 intersects the y-axis at a point equal to 1/Smax and the slope of the resulting straight line is equal to Kd/Smax. Kd/Smax divided by 1/Smax gives the required Kd value.

S2. Determination of the kinetic dissociation constant

If the concentration of the competing antigen is many times greater than the concentration of the immobilized one, the dissociation of the antibody – immobilized antigen complex becomes practically irreversible, and the approximation of an irreversible monomolecular reaction can be used to describe it:

AbAg → Ab + Ag.

The kinetics of irreversible dissociation of the antibody – immobilized antigen complex is described by the equation:

$$\frac{d[AbAg]}{dt} = -\,kd[AbAg] \qquad (7).$$

As a result of dividing both parts of the equation (7) by [AbAg] and multiplying them by dt, the following equation is obtained:

$$\frac{d[AbAg]}{[AbAg]} = -\,kd\;dt \qquad (8).$$

After integration, the following equation is obtained:

$$\ln[AbAg] = -\,kd\;t + Const \qquad (9).$$

From the boundary conditions (at t=0 [AbAg]=[AbAg]0, where [AbAg]0 is the concentration of the complex before adding a competitor), Const=ln[AbAg]0.

Using the properties of logarithms, the following equation is obtained:

$$l\,n\frac{[AbAg]}{[AbAg]_0} = -\,kd\;t \qquad (10).$$

The [AbAg]/[AbAg]0 ratio is the fraction of the immune complex that is not destroyed after the dissociation over time t. This ratio is equal to the ratio of analytical signals S/S0 (S0 is the signal before the addition of a competitor and S is the signal after incubation with a competitor during time t). Thus, it is easy to obtain the value of the kinetic dissociation constant kd from the ratio of the analytical signals and the t value using equation (10).

S2. Comparison of multiplex and monoplex LFIA

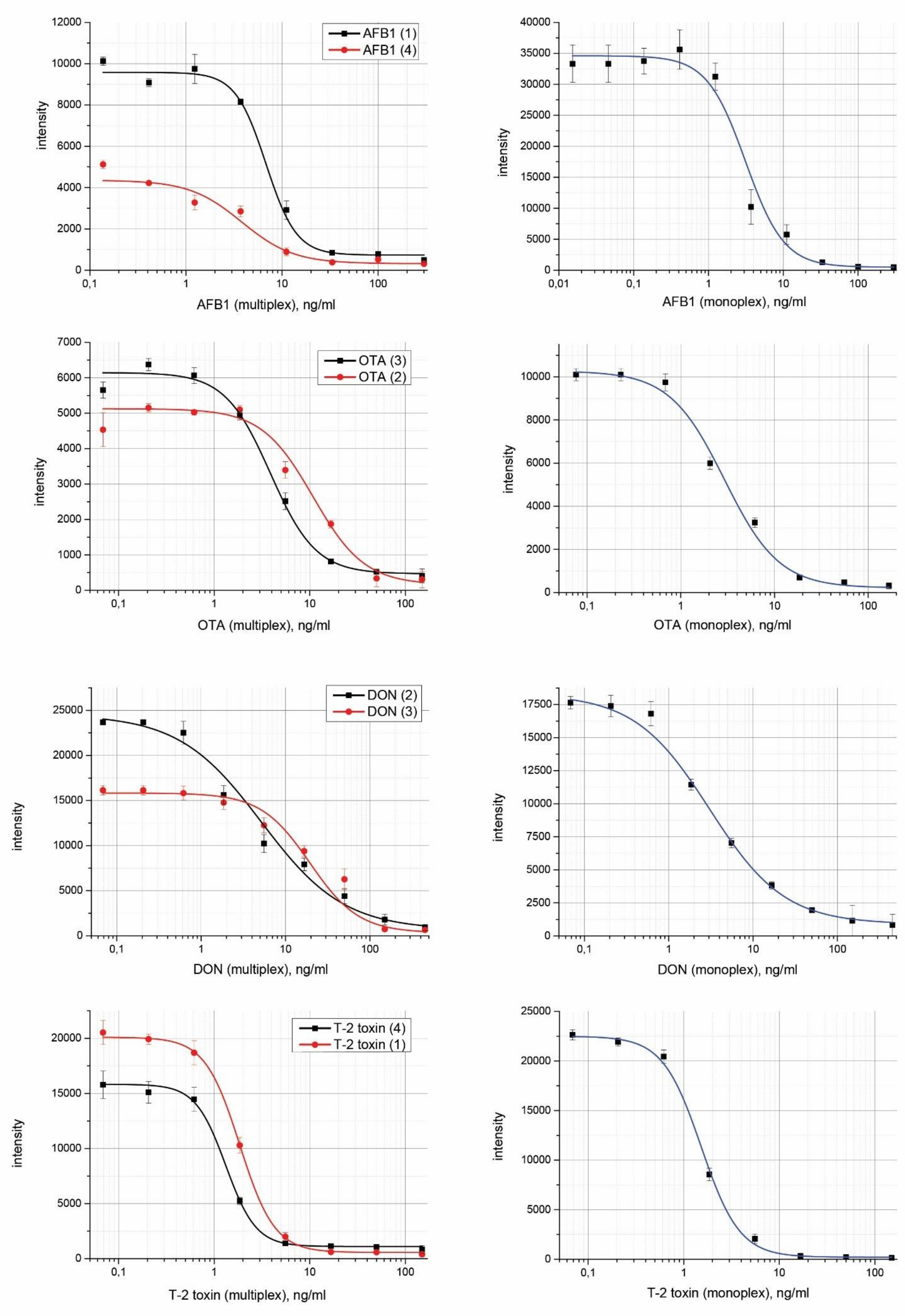

Figure S1. Calibration curves for mycotoxins in the multiplex LFIA. The test strips are arranged from right to left according to the AFB1 concentrations of 300, 100, 33.33, 11.11, 3.70, 1.24, 0.41, 0 ng/mL; DON concentrations of 450, 150, 50, 16.67, 5.56, 1.85, 0.62, 0 ng/mL; OTA concentrations of 150, 50, 16.67, 5.56, 1.85, 0.62, 0.21, 0 ng/mL; T-2 toxin concentrations of 150, 50, 16.67, 5.56, 1.85, 0.62, 0.21, 0 ng/mL. The experiments were implemented in triplicate, and standard deviations are given as error bars.

S3. Complex dissociation kinetics

To check the influence of the incubation time of the dissociation of immune complexes, we have registered these processes under high excess of the competitor (free antigen). The time used in our study accords to the interval with rapid decrease of immune complexes quantity for all four studied mycotoxins.

As a coating antigen in the ELISA, a hapten-protein conjugate (1 μg/mL, 100 μL) in PBS was added to the microplate wells and incubated for 2 h at 37°C. After washing three times in PBST, 1% BSA solution (100 μL) in PBST was added and incubated for 1 h at 37°C. After washing, specific antibodies (100 ng/mL, 50 μL) in PBST were added. Then, goat anti-mouse antibodies labeled with horseradish peroxidase (2 μg/mL, 50 μL) were added and incubated for 1 h at 37°C. After that, the microplate was washed. To compare the signal, an excess amount of the antigen (0.5 μg/mL) was added to half of the microplate wells and incubated for 1 h at 37°C. To measure the peroxidase activity, a substrate solution (0.42 mM TMB and 1.8 mM H2O2 in 0.1 M citrate buffer (pH 4.0), 100 μL) was added to the microplate wells. After incubation (~1 h) at room temperature, the reaction was stopped by adding 1 M H2SO4 (50 μL). The optical density of the reaction product was measured at 450 nm using a Zenyth 3100 microplate photometer.

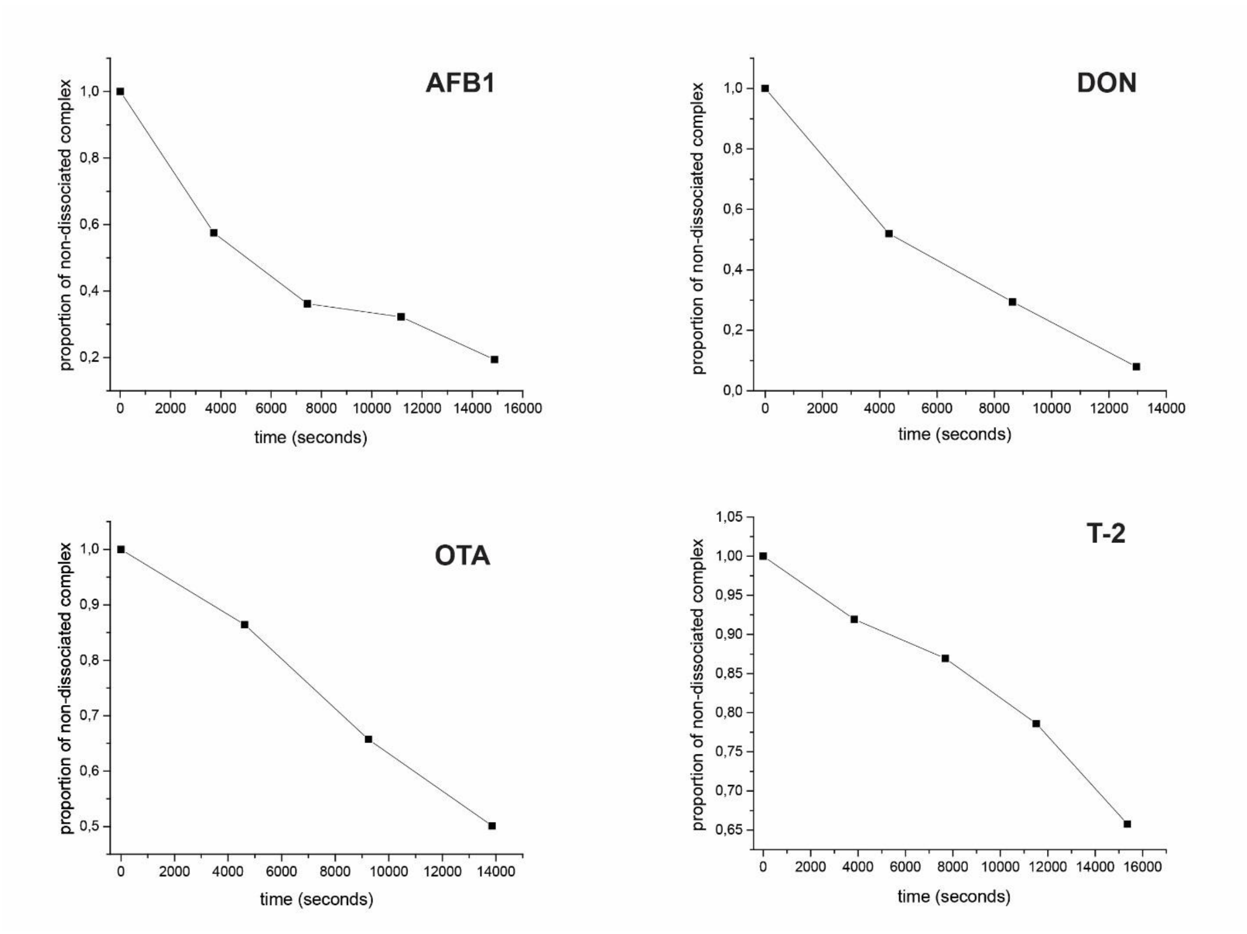

Figure S2. Graphs for mycotoxins in the dynamics of dissociation of the immune complexes in the presence of a multiple excess of a competitor.

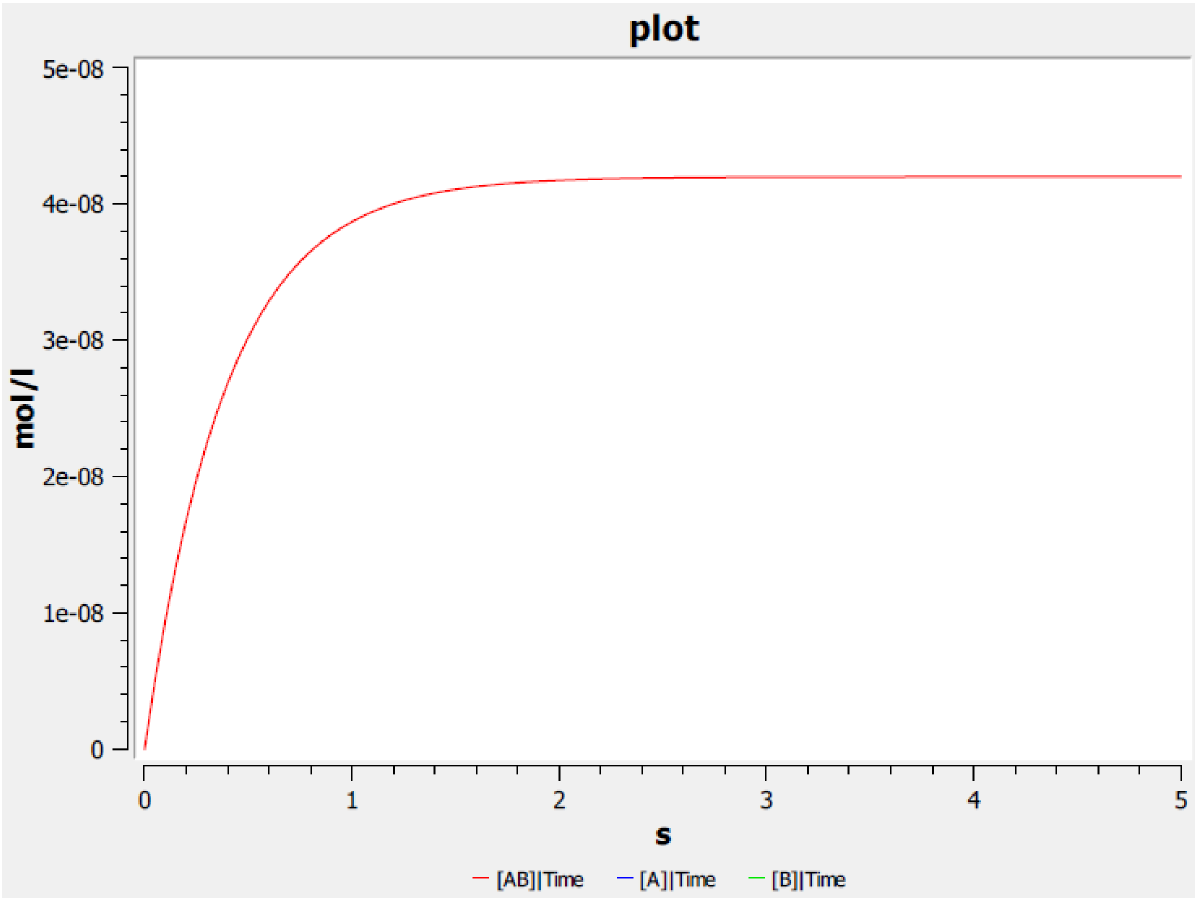

Figure S3. Kinetics of approaching equilibrium of the anti-AFB1 antibody reaction with the AFB1-BSA conjugate.
